# The Unsung Heroes: The Profile of the Donor at a Southern Italian Milk Bank and Driving Factors in Human Milk Donation

**DOI:** 10.3390/children11121502

**Published:** 2024-12-10

**Authors:** Pasqua Anna Quitadamo, Laura Comegna, Federica Zambianco, Giuseppina Palumbo, Massimiliano Copetti, Maria Assunta Gentile, Antonio Mondelli, Isadora Beghetti, Luigi Corvaglia

**Affiliations:** 1 Human Milk Bank, NICU Ospedale “Casa Sollievo della Sofferenza”, 71013 San Giovanni Rotondo, Italy; comegna@operapadrepio.it (L.C.); palumbogiuseppina@tiscali.it (G.P.); ma.gentile@operapadrepio.it (M.A.G.); a.mondelli@operapadrepio.it (A.M.); 2 San Raffaele Faculty of Medicine, University of San Raffaele Vita-Salute, 20132 Milano, Italy; bancadellatte@operapadrepio.it; 3 Statistical Department, Casa Sollievo della Sofferenza, 71013 San Giovanni Rotondo, Italy; m.copetti@operapadrepio.it; 4 Neonatal Intensive Care Unit, IRCCS Azienda Ospedaliero-Universitaria Bologna, 40138 Bologna, Italy; isadora.beghetti@unibo.it (I.B.); luigi.corvaglia@unibo.it (L.C.)

**Keywords:** human milk donors, milk donation, human milk bank

## Abstract

Background/Objectives: One of the most effective strategies to mitigate morbidity associated with preterm birth is the use of human milk. The first choice is the mother’s milk; if that is not available, human milk donated to milk banks is the second choice. The recruitment of milk donors is essential for enhancing the effectiveness and efficiency of donation. This study aims to profile the donors of a southern Italian milk bank, examine how maternal and neonatal factors impacted the volume and duration of donation and analyze the trend over the last ten years. Materials and Methods: Data were collected from the milk bank database and hospitalization medical records, encompassing 593 donors and 13 years of activity of the Human Milk Bank from 2010 to 2022. Several variables were assessed: maternal characteristics—maternal age, employment status and the type of profession; pre and perinatal characteristics—type of delivery, parity, previous breastfeeding experience, birth weight and gestational age; milk donation characteristics—volume of milk donated and duration of donation. The trend of the characteristics was studied over time. Statistical correlations were conducted to assess the relationships between variables and the volume and duration of the milk donation. Results: In our cohort of donors, the most prevalent category consists of women over 30 years of age who are multiparous, have prior breastfeeding experience, are workers and have given birth to full-term babies. Maternal age and gestational age significantly influence both the volume and duration of milk donation. The type of delivery and occupation impact the volume of donated milk. There has been a decline in the number of donors over time. However, the trends in both the quantity and duration of milk donations have remained stable over the past decade, with the exception of the year immediately following the COVID-19 pandemic. Conclusions: We have outlined the prevailing average profile of the milk donor to a milk bank in Southern Italy. Factors impacting the volume and duration of donation, such as maternal age, occupation, type of delivery and gestational age, were identified. The volume and duration of donations have remained largely stable, with the exception of 2021, when the pandemic significantly reduced milk donations to the milk bank.

## 1. Introduction

Technological advancements in the field of biology have allowed for great discoveries on the beneficial properties of breast milk that, however, do not fully explain the functions of the countless elements present in the various stages of lactation, which act both individually and in combination with others, in a biological concert of health. However, the unmatched value of breast milk for babies has never been clearer. The benefits of breast milk on health are particularly significant for the most vulnerable category of newborns, which are premature babies, especially those with low birth weight [1,2,3]. The first choice for preterm infant nutrition is the mother’s own milk. If it is not available, donated human milk from milk banks is recommended [1].

Donor recruitment represents the first and most important phase of the human milk banking process; understanding the characteristics of women who donate and how these factors influence both the quantity of milk donated and the duration of donation can provide valuable insights. This knowledge can ultimately enhance the overall donation system and ensure that more infants receive the benefits of human milk (HM). This topic has been extensively studied in relation to other forms of donation, such as blood donation [4], yielding positive results. We believe that this model can also be applied to human milk donation, which is equally virtuous. Little is known about how the characteristics of milk bank donors evolve over time and how these changes may impact the donation [5,6,7,8,9,10,11].

Several authors [5,6,7,8,9,10,11,12,13] on the topic have emphasized the critical role of donated human milk as a recommended alternative for feeding premature or low birth weight infants in the absence of sufficient maternal breast milk. They highlight the necessity of accurately characterizing milk donors to promote donations to human milk banks. According to Gutierrez Dos Santos [12], various demographic characteristics have been consistently documented across different regions, while other aspects, such as gender and ethnicity, have received less focus. Furthermore, the elements affecting the composition of donated breast milk—including the timing of delivery, the type of milk and the maternal dietary intake—have not been thoroughly examined. The authors also highlight significant research gaps, such as the motivations and obstacles related to milk donation, the historical context and practices surrounding breastfeeding, the factors that inspire donors to express and store excess milk and the trends in milk donation.

A systematic review by Kaech and Kilgour [14] evaluated literature published until December 2021, involving 7053 participants. Their analysis focused on donor characteristics, including socio-demographic factors and milk donation history, as well as neonatal aspects, such as gestational age and birth weight. Another prominent systematic review by Gutierrez Dos Santos [12] synthesized literature available up to August 2020, covering a diverse range of geographical contexts, including the United States, Brazil and various countries across Europe, Korea and China. Other studies [5,15,16] have been conducted as single-center retrospective cohort studies, analyzing data from medical records and databases. Notably, Jarmoc et al. [15] conducted a nine-year study involving mothers who donated to the Mothers’ Milk Bank Northeast in the United States until 2019. Similarly, Nangia et al. [16] evaluated the demographic profiles of 1553 milk donors over a 21-month period. An Italian study by Bocci et al. [5] assessed 304 donors from 2010 to 2017, focusing on socio-demographic factors, pregnancy and childbirth-related variables, parity, prior donations (including blood) and the volume and duration of milk donations.

Additionally, Anitha et al. [6] conducted a six-month investigation in a tertiary NICU, collecting data on the prevalence of human milk donors and the characteristics of regular donors and their newborns from a sample of 864 mothers. Tran led a retrospective survey [7] of Vietnam’s first human milk bank, which recruited 517 donors up to 2023, to assess the characteristics and factors associated with higher donation volumes. Similarly, Novoselac [13] analyzed a sample of 200 women during the first three years of the Croatian human milk bank’s operation, concluding their study in 2023. Sierra-Colomina [10] investigated the relationship between social and demographic donor variables and neonatal variables in relation to the volume of human milk delivered using a sample of 415 donations from the Hospital Doce de Octubre HMB over a four-year period from 2009 to 2013. Osbaldiston [8] and his team used a telephone interview tool administered to 87 donors and 19 non-donor controls to collect descriptive information not only on demographics but also on aspects as diverse as lifestyle, emotional aspects, motivations and the different challenges and obstacles for breastfeeding and donation and they also explored potential correlations with the amount of milk donated. In a cross-sectional study conducted by Meneses [9] in 2013 in Rio de Janeiro, Brazil, involving 695 mothers of infants under one year of age from nine primary health care units with human milk donation services, the findings indicated that greater volumes of donated milk are associated with longer donation durations, earlier initiation of donation, prior donation experience, lower maternal and gestational ages [14].

The study of Kaech [14] underscores that promoting early donation and focusing recruitment efforts on mothers of premature infants may enhance donation duration and increase milk volumes. Jarmoc from North America [15] indicated an increasing number of donors over time despite a decline in those likely to have premature infants. Conversely, Nangia’s study [16] in a developing country revealed that the majority of donors were younger and more frequently had a history of premature or low birth weight births, with many having newborns admitted to the NICU. Additionally, Bocci’s research in Siena [5] highlighted a superior socio-economic profile among Italian donors compared to non-Italian donors, who were found to be at a higher risk of preterm births. Notably, the duration of donation emerged as a significant predictor of the volume of milk donated, and the authors concluded that mothers of preterm infants possess a heightened awareness of the critical importance of milk donation and nutrition for the health of vulnerable infants.

Sierra-Colomina [10] documented the link between prior donations, lower gestational age of infants and early initiation of milk donation, with an increase in volumes of milk donated to human milk banks. Anitha in South India [6] revealed that educated, socioeconomically advantaged pluriparous donors contributed significantly more to the outcomes of the HMBs. Similarly, research in Vietnam [7] found that mothers with higher educational attainment were more likely to donate breast milk for extended periods than those with lower education levels. The authors stressed the importance of protective measures, promotional initiatives and breastfeeding support, especially for mothers of premature or ill infants, to ensure they can adequately provide breast milk and have a surplus for donation. This sentiment is echoed by Meneses in Brazil [9], who confirmed that promoting breast milk donation and offering information and support from healthcare professionals in primary health units are essential for facilitating human milk donation practices.

This study aimed to define the typical profile of milk donors to characterize mothers who donate milk to a milk bank in Southern Italy. We also explore trends in donor characteristics and evaluate the associations among maternal and neonatal factors and milk production.

## 2. Materials and Methods

Data have been collected from the milk bank database and from the hospitalization medical records and included the mother’s age, profession, type of delivery, gestational age, birth weight, parity, prior lactation experience, milk volume donated and duration of donation. Women who donated to the milk bank between 2010 and 2022 were included in the study. Exclusion criteria consisted of women with insufficient data for analysis. A total of 593 donors were included in the study. We notified the ethics committee of the start of this observational retrospective study: no formal approval was needed for this type of study. Written informed consent was obtained for anonymized patient information to be published in this article. The study examined several variables, which can be categorized into three main groups: maternal characteristics, pre and perinatal characteristics and milk donation characteristics.

*Maternal Characteristics*: maternal age, employment status (presence or absence of a job) and the type of profession. *Pre and perinatal characteristics*: type of delivery, parity (primiparous or multiparous), previous breastfeeding experience, birth weight (BW) (in grams) and gestational age (GA) expressed in weeks, with infants born at less than 37 weeks classified as premature. *Milk donation characteristics*: the volume of milk donated (expressed in milliliters) and the duration of donation (expressed in days) were assessed.

Certain variables were also categorized into groups for analysis. Maternal age was divided into five categories: ≤25 years, 26–29 years, 30–35 years, 36–40 years and >40 years. GA was categorized into five groups (≤29 w, 30–36 w, 37–41 w, >41 w); BW was categorized into five groups: ≤1500 g, 1501–2500 g, 2501–3500 g, 3501–4000 g and >4000 g. The volume of donated milk was grouped into five categories: ≤1000 mL, 1001–5000 mL, 5001–10,000 mL, 10,001–50,000 mL and 50,000–100,000 mL.

Statistical correlations were performed to assess relationships between maternal age, employment status, parity, previous breastfeeding experience, gestational age and birth weight and the two characteristics of milk donation: volume and duration. Additionally, trends over time were analyzed for maternal age, previous breastfeeding and employment status, as well as for the quantity and duration of milk donations. The trend in the number of donors per year was compared with the number of births each year, expressed as a percentage of donors relative to the number of women who gave birth. This adjustment provides a clearer perspective on the donor population size, given that the number of women donors in a single center is directly influenced by the number of births at that center.

Baseline demographical and clinical characteristics were reported as median and inter-quartile range for continuous variables and as frequency and percentage for categorical variables. Normal distribution assumption was checked using the Shapiro–Wilk test by graphical inspection of the QQ-plot. Continuous variables were compared between groups using the Kruskall–Wallis test or Mann–Whitney U-test as appropriate. Correlations between continuous variables were assessed using the Spearman correlation coefficient. A *p*-value < 0.05 was considered statistically significant. All analyses were carried out using R software Version 4.4.1.

## 3. Results

**Figure 1:**
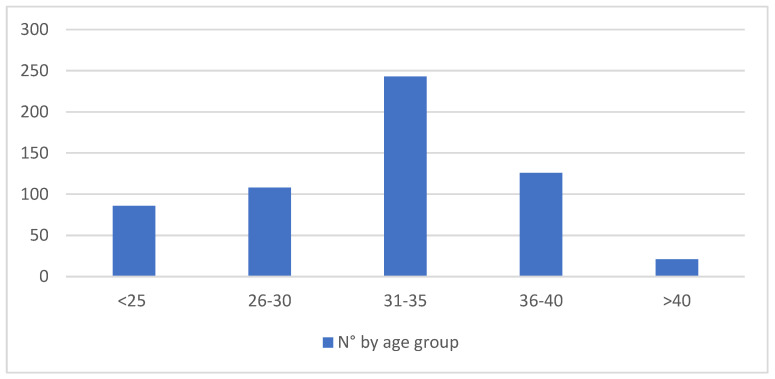
Distribution by maternal age group. Representation of age variable categorized by age groups. The categories represent the numerical sample of donors for each individual age group across the years.

The descriptive results were as follows. *Maternal Characteristics*: (Table 1, Figure 1) The mean age of the participants was 31.38 years, with a median of 32 years. The age group most represented was between 31 and 35 years.

**Figure 2:**
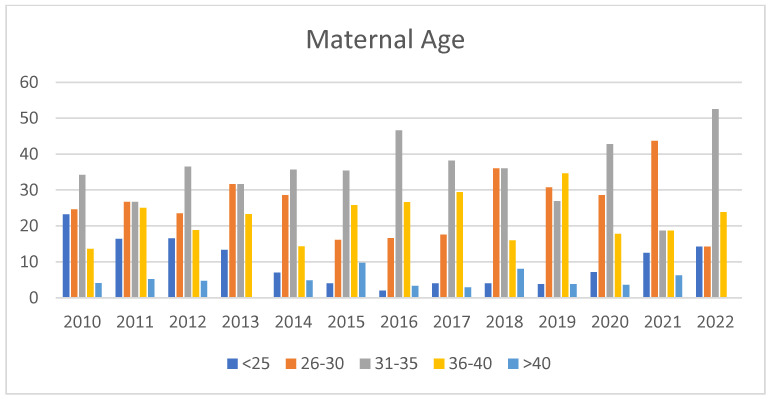
Annual Trend of Donor Age. Trend of the age variable categorized into age groups. The data for age groups are expressed as percentages corresponding to individual years.

The prevailing demographic was aged between 31 and 35 years in eight out of the thirteen years analyzed, and it was equal in number to the 26 to 30 age group in another three years (Figure 2).

**Figure 3:**
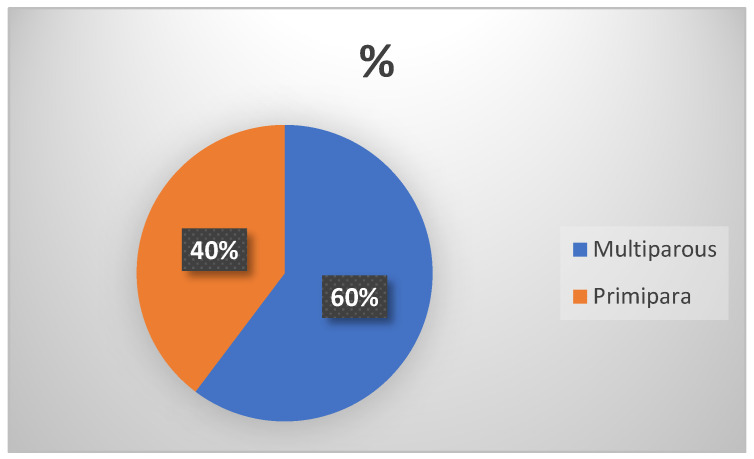
Parity. Percentage of women in the categories of primiparous and multiparous.

Sixty percent of the women who donated were multiparous, while forty percent were primiparous (Table 1, Figure 3).

**Figure 4:**
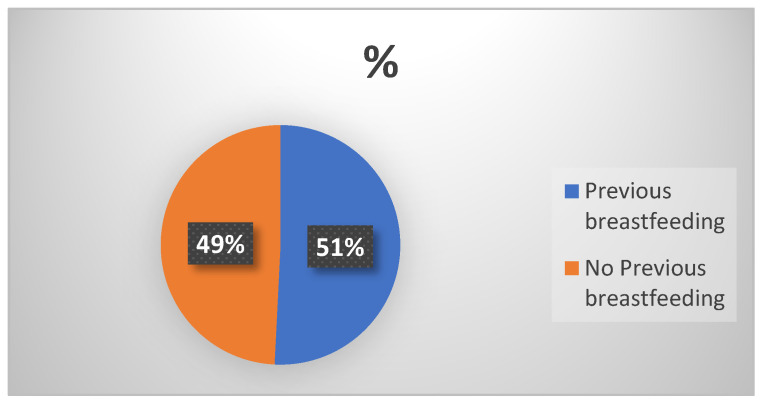
Previous breastfeeding. Data expressed as the percentage of women with previous breastfeeding experience compared to those without.

For previous breastfeeding (Table 1), 51% of donors had previous breastfeeding, and 49% did not (Figure 4). The trend over time sees the highest percentage of no-previous breastfeeding in 2010 (83%) and the lowest in 2017 (27%).

**Figure 5:**
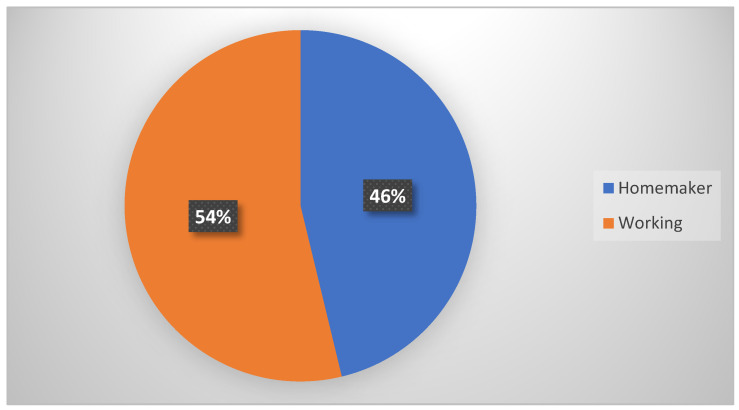
Occupation. A total of 54.3% of the donors were working, compared to 46.7% who were housewives.

For occupation (Table 1, Figure 5), 322 donors had jobs, whereas 271 were homemaker mothers.

**Figure 6:**
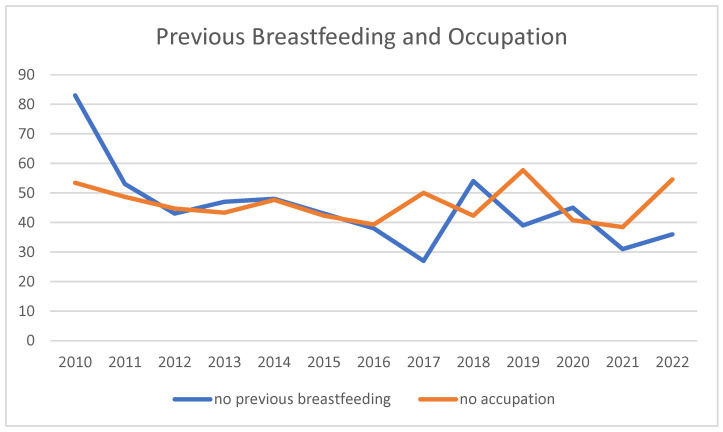
Previous breastfeeding and employment. Trends over the years of the variables expressed as the percentage of women who did not previously breastfeed and who are not working in the annual population of donors.

Over the years, there has been an increase in the percentage of women aged over 35 and housewives within the donor population (Figure 2), alongside a decrease in women with no previous breastfeeding experience (Figure 6). The lowest value of the percentage of unemployed was 39.29% in 2016, and the highest was 57.69% in 2019, while the 2022 figure for the unemployed was 54.55% (Figure 6).

**Figure 7:**
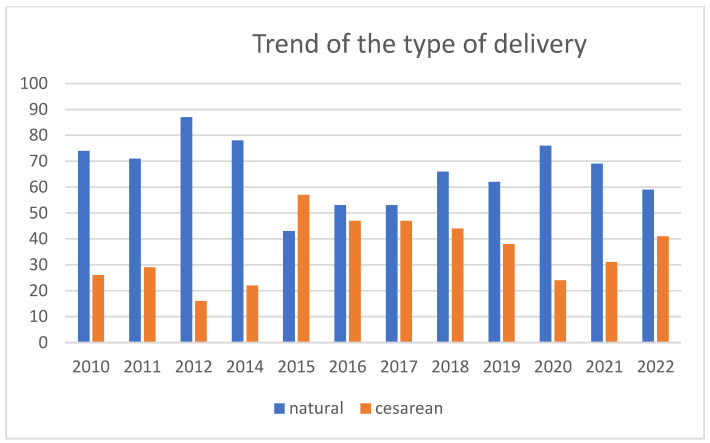
Percentage distribution of natural births and cesarean sections over the specified period.

*Characteristics of Childbirth*: For the type of delivery (Table 1), a total of 68.3% of deliveries were spontaneous, while 31.7% were delivered via cesarean section, and the trend over time showed an increase in the percentage of donors who had a cesarean section (between 2015 and 2019, between 38% and 57% of the total number of deliveries were cesarean) (Figure 7).

For gestational age (Table 1), 15.3% were classified as premature, 81.1% as full-term and 3.7% as post-term (>42 weeks). Infants with a birth weight of less than 2500 g constituted 15.7% of the population, with those weighing less than 1500 g (very low birth weight, VLBW) accounting for 6.1% (Table 2).

*Characteristics of Donation* (Table 1): The average volume donated was 5182 L, with a median of 2100 L (Table 1). The mean duration of the donation period was 73.44 days, and the median was 60 days (Table 1). The mean volume donated was 5555.99 L for infants born at >32 weeks and 13,386.98 L for infants born at ≤32 weeks (Table 3). The mean volume was 15,336.18 L for infants weighing <1500 g, 5104.36 L for infants weighing 1500–2500 g and 5622.63 L for infants weighing >2500 g (Table 3).

**Figure 8:**
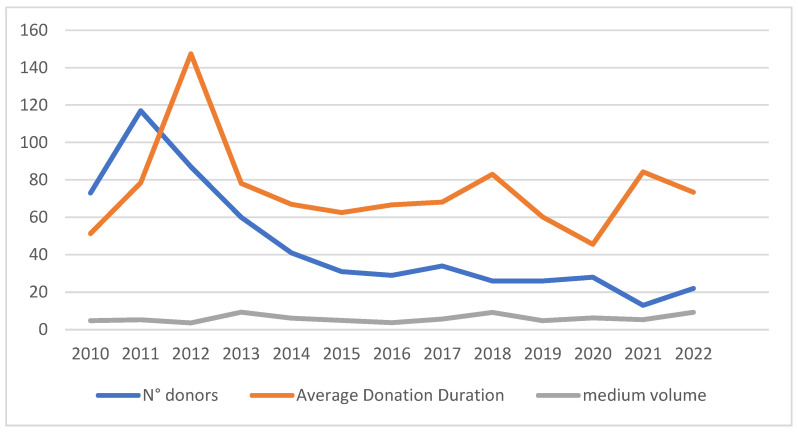
Trends over the years in donation characteristics: number of donors, average donation duration (in days) and average volume donated (in mL).

Notably, the average amount of milk donated remained consistent throughout the study period. Although the number of donors was very high during the first year of the bank’s operation, it decreased in the following two years before stabilizing, with the exception of a notable decline observed in 2021, in the post-COVID period (Figure 8).

**Figure 9:**
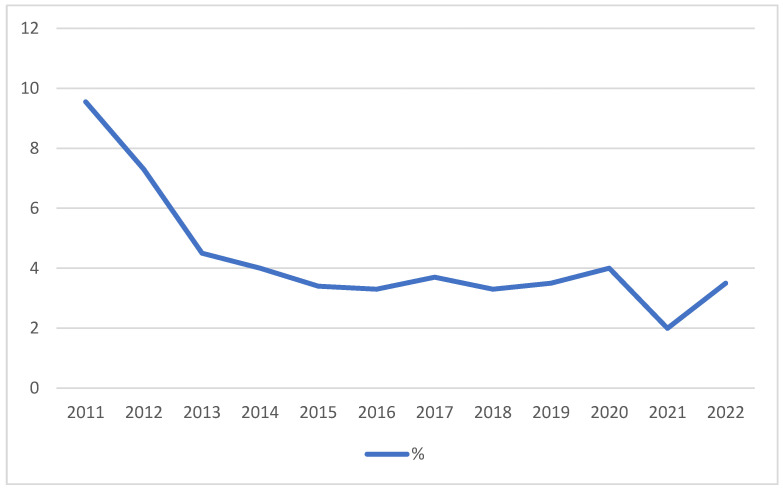
Annual trend of donor percentage among women who gave birth in the San Giovanni Rotondo.

The trend in the annual number of donors was analyzed in relation to the annual number of births, expressed as a percentage of donors relative to the total number of women who gave birth (Figure 9).

Donors who had cesarean sections contributed 17.4% more in total volume but did so over a duration that was 6% shorter.

Table 4 highlights correlations between demographic factors and donation characteristics. Occupation and type of delivery significantly correlated with the amount of milk donated. Previous breastfeeding experience had a notable impact on the duration of donation. A strong correlation was observed between maternal age and both the duration (*p* < 0.0001) and quantity (*p* < 0.0001) of milk donated. Gestational age demonstrated significant correlations (*p* < 0.0009 to *p* < 0.0008) with donation outcomes. Birth weight was also found to have a statistically significant impact on the duration of milk donation.

## 4. Discussion

Human milk mitigates the adverse effects of premature birth and should be the standard of care in all NICUs [17,18,19,20,21]. The first choice should always be a mother’s own milk, followed by donated milk as a secondary option [20,21,22,23,24,25,26]. In the nutrition of the most vulnerable infants, the utilization of donated human milk [18,27] appears to be a strategic approach, enabling these infants to avoid formula feeding while awaiting adequate milk production from their mothers. Banked human milk, despite its differences from fresh breast milk due to heat treatment (pasteurization), contains species-specific bioactive substances that provide significant biological functions and protective effects for the immature infant, even if it is to a lesser extent than fresh breast milk [28].

Recent meta-analyses [22,23,29,30] and individual studies indicate that the use of human milk (maternal milk and/or donor milk) compared to formulated milk (FM) significantly reduces the overall incidence of NEC (necrotizing enterocolitis) by 50% to 75%. Furthermore, the risk of surgical NEC is also markedly diminished [19,22,23]. The protective effect of HM against NEC is dose-dependent [19,22]. In the critical period of the first 14–28 days, the administration of FM should be avoided to ensure that vulnerable infants receive a diet exclusively based on human milk [19,20]. To effectively mitigate the risk of NEC, all very low birth weight infants should exclusively receive human milk (either mother’s milk or donor human milk) from birth until 36 weeks of post-conceptional age [18,21]. Exclusive feeding with HM may also lower the incidence of late-onset sepsis (LOS) [22,24], severe retinopathy of prematurity (ROP) [22,24] and BPD [22,24]. The reduction in NEC incidence, coupled with the faster achievement of full enteral feeding made possible by human milk utilization, contributes to decreased lengths of hospitalization [20,25,26,29,31]. Nevertheless, our clinical experience within the NICU supports this approach [32]. Therefore, an important benefit of DHM (donor human milk) includes the “prevention” of taking formula milk.

Globally, there has been a significant increase in the number of human milk banks [33]. In 2020, over 800,000 vulnerable infants received DHM from HMBs; conversely, approximately 500,000 infants born at less than 32 weeks of gestation lacked access to DHM [34]. Therefore, it is crucial to identify strategies that can enhance the supply of DHM. In Italy, some regions have achieved total coverage of the need for human milk thanks to a well-established network of human milk banks, which harmonizes the collection of donated milk with demand. However, in other regions, access to human milk for premature infants is limited, with supply falling short of actual needs. In Puglia, the availability of human milk for premature infants in NICUs is less than 10%, primarily due to the lack of a systematic connection between the two existing milk banks and the obstetrician departments and neonatal intensive care units throughout the region. Our milk bank, established in 2010, is located in the southern region of Italy, which currently holds the record for the highest number of active milk banks in Europe, with a total of 44. However, it is important to note that the majority of these banks are concentrated in the northern and central–northern regions of the country. The predominant profile of the donors at our milk bank, “Allattiamolavita”, as revealed by the study, is a woman aged between 31 and 35 years, who is a worker, multiparous, has previously breastfed and has given birth to a full-term or near-term newborn. Conversely, in developing countries, donors at human milk banks are often younger women who have given birth to premature or low birth weight infants [16]. In Brazil [9], the average donor profile consists of very young women, who are often multiparous homemakers. In the United States, the Human Milk Banking Association of North America (HMBANA) reports a donor demographic that predominantly consists of older, educated, and married women [15].

The *age* of donors is one of the most studied variables in the literature. Findings from India [6] indicate that younger women, with an average age of 21.6 years, are more likely to donate. In Brazil, data suggest that 60% of donors are under the age of 25. In France, the average age of donors is reported to be 28.5 years, followed by Italy at 30.8 years and Poland, where the average age ranges from 31 to 33 years [12]. In our study, the average age of donors is 31.3 years, with the predominant age group being 31 to 35 years, followed by those aged 36 to 40 years and those under 25 years. This trend indicates a significant increase over time in the proportion of older women donors, while the proportion of younger donors has significantly decreased, and since 2015, the primary demographic of donors has been women over the age of 35. We believe that this trend could suggest that advancing age may be linked to an increased awareness of the significance of proper nutrition for vulnerable infants. In addition, donors over the age of 35 tend to make larger average donations, indicating that informed awareness and conscious motivation are key factors driving effective donation [35].

With respect to *parity*, 60.3% of our sample of donors are multiparous, which is consistent with Olbaldiston’s findings [8]. This could be attributed to a greater familiarity with breastfeeding gained from previous experiences. However, this trend has not been universally confirmed in other studies.

In terms of *occupation*, 46.7% of donors are homemakers. Bocci’s case study in Siena [5] indicates that the majority of donors are working, with 73% in stable jobs and 27% unemployed, highlighting socio-economic differences between women from northern and southern Italy. While occupation was statistically significant in influencing the volume of donations, it did not affect the duration of donation. Working donors outnumber self-employed women, with 45% versus 12.5%, respectively. In our region, the lack of facilities and policies supporting women with infants and young children, particularly self-employed professionals and traders, represents a risk factor not only for milk donation but also for breastfeeding.

Regarding *previous breastfeeding* experience, just over half of the donors reported having such experience, which is lower than the 60% parity rate. Gutierrez’s review [12] emphasizes the lack of data on breastfeeding history, noting that only two studies have addressed this topic. This gap highlights the need for future research to focus on this important aspect, as well as on supporting breastfeeding and milk extraction practices. Such research could better elucidate the pathways leading women to produce excess milk for donation.

Apropos the *type of birth*, it is commonly believed that women who have a vaginal delivery experience an easier time breastfeeding due to early contact with their babies and quicker physical recovery. Bocci’s experience in Siena [5] indicated that donors who had a natural birth donated for a longer period but at a smaller volume compared to those who had a cesarean delivery. In our case study, donors who underwent cesarean sections donated an average of 17.4% more milk volume compared to those who had vaginal deliveries, and the type of delivery was statistically correlated with the quantity of milk donated. Our hospital’s particular emphasis is on implementing best practices to promote breastfeeding among women who deliver via cesarean section. Therefore, we believe this finding is significant as it suggests that, with optimal support and interventions, the anticipated gap in breastfeeding effectiveness between women who give birth naturally and those who have cesarean sections could be minimized or even reversed. Nangia’s study [16] on donors from developing countries found that multiparous women who had natural births predominated over primiparous women with cesarean deliveries. Conversely, the Brazilian study by Meneses [9] indicated that neither the type of delivery nor parity were distinguishing factors between donor and non-donor women.

Our findings indicate that the highest volume of milk donated comes from mothers of premature and low birth weight infants, and this trend has been consistent in our experience [11] and is corroborated by previous analyses conducted by Sierra Colomina in Spain [10], Nangia in India [16], Jarmoc in Massachusetts [15], Bocci in Italy [5] and Novoselac in Croatia [13]. In these studies, donor mothers of preterm infants consistently donated larger volumes of milk, demonstrating a statistically significant difference. Notably, in the Indian cohort identified by Nangia [16], 62.3% of the total volume of donated milk was contributed by mothers of premature infants. In Gutierrez’s review [12], the percentage of mothers of preterm infants who become milk donors ranges from 8% to 24%, and in two studies conducted in India and Brazil [12,16], this figure can reach as high as 50%. Proximity to a NICU has been identified as a facilitating factor for milk donation [33], and the limited milk intake by very small premature infants during their first weeks of life often leads to milk accumulation. In addition, mothers may have greater logistical access to milk expression and storage facilities. However, the association between mothers with infants in the NICU and the ease of milk donation is not uniformly reported [36]. Conversely, the stress associated with an unexpected and emotionally charged event, such as premature birth, may negatively impact milk production [36]. Nonetheless, we believe that for mothers of premature infants, the opportunity to provide a critical health resource like their own milk during a time when their child’s care is managed by NICU staff—rather than by themselves, as they had hoped—can serve as a strong incentive for breastfeeding and milk expression. Regular presence in the NICU allows these mothers to recognize the importance of feeding vulnerable newborns, thereby enhancing their understanding of the value of milk donation.

The average volume of donated milk was 5181.32 mL, which is consistent with the literature, with the exception of Nangia’s study [16], which reported lower mean volumes, and Jarmoc’s study, which reported higher volumes. It is important to note that all donors were included in this analysis. This inclusion reflects our principle that “no drop of milk is wasted”, particularly since small quantities often consist of colostrum, which is highly valuable. Analysis of longitudinal trends indicates a pattern characterized by an initial surge in 2011, marked by a significant number of donors contributing over extended durations. Subsequently, there has been a noted decline in the number of active donors, which should spur us to increase our commitment to promoting donations, particularly among categories that are at a higher risk of not donating. Our efforts should begin with the promotion of breastfeeding, as breastfeeding and milk donation mutually enhance one another [37].

Interestingly, during 2020, the year of the pandemic, the number of donors remained stable, contrary to global trends [35], while the exception was 2021, which saw a drastic reduction in donations due to the post-COVID effects. This represents another negative consequence of the pandemic, which also impacts this specific group of patients [35,38]. This figure may serve as a warning for future pandemics, highlighting the urgent need for a current prevention protocol to safeguard human milk donation programs. This protocol should encompass all necessary measures to prevent any potential disruption of these essential services.

We believe that our study contributes to the body of research aimed at increasing the availability of human milk for some of the most vulnerable infants. By improving our understanding of donor characteristics, we can enhance the effectiveness of milk donation promotion efforts. The limitations of this study include its monocentric design, which reflects a localized reality, and the omission of an exploration into the motivations behind milk donation. Future research should adopt a multicenter approach to encompass a broader geographical scope and develop a comprehensive profile of human milk donors.

## 5. Conclusions

In our cohort of human milk donors, the predominant profile comprises women over 30 years of age who have experienced multiple childbirths, possess breastfeeding experience, are workers and have had full-term pregnancies. Both maternal age and gestational age significantly influence the quantity and duration of milk donation. Occupation and type of delivery significantly correlate with the amount of milk donated. Previous breastfeeding experience had a notable impact on the duration of donation.

We have observed a gradual decline in the number of donors over time while the volume and duration of milk donations remained stable over the past decade, with the exception of a notable decrease following the COVID-19 outbreak. This trend should serve as a warning of the potential impact of future pandemics on milk donation rates.

## Figures and Tables

**Table 1:** Data table. Maternal, perinatal, neonatal and donation variables expressed as mean and as median and interquartile range for continuous variables and as frequency and percentage for categorical variables.

	N = 593
**Maternal Age**	
Mean (SD)	31.38 (5.68)
Median (Q1, Q3)	32.00 (28.00, 36.00)
Min–Max	17.00–47.00
**Occupation**	
No	271 (45.7%)
Yes	322 (54.3%)
**Birth Weight**	
Mean (SD)	3114.65 (719.49)
Median (Q1, Q3)	3250.00 (2890.00, 3540.00)
Min–Max	430.00–4530.00
**Type of delivery**	
Cesarean	184 (31.7%)
Spontaneous	396 (68.3%)
**Gestational age**	
Mean (SD)	38.40 (3.44)
Median (Q1, Q3)	40.00 (38.00, 40.00)
Min–Max	20.00–43.00
**Parity**	
Multiparous	324 (60.0%)
Primipara	216 (40.0%)
**Previous breastfeeding**	
No	292 (49.2%)
Yes	301 (50.8%)
**Volume of donated milk**	
Mean (SD)	5181.32 (9476.93)
Median (Q1, Q3)	2100.00 (900.00, 5200.00)
Min–Max	23.00–97,760.00
**Duration of donation**	
Mean (SD)	73.44 (54.94)
Median (Q1, Q3)	60.00 (30.00, 112.75)
Min–Max	1.00–243.00

**Table 2:** Perinatal variables, including type of delivery, birth weight and gestational age, expressed as percentages of the various categories.

Type of Delivery		Birth Weight		Gestational Age	
	%	(g)	%		%
Spontaneous	68.3	<1500	6.1	≤29	4.3
Cesarean	31.7	1501–2500	9.6	30–36	10.9
		2501–3500	58	37–41	81.1
		3501–4000	23.1	>41	3.7
		>4000	4.2		

**Table 3:** Donated volume of milk. Number and percentage of donors categorized by the volume of donated milk, expressed in milliliters. Volume distribution by GA and BW expressed in liters.

Volume mL	N°	%	GA and BW	Mean Volume Liters
≤1000	172	29.1	>32 weeks	5555.99
1001–5000	264	44.7	≤32 weeks	13,386.98
5001–10,000	80	13.5	<1500 g	15,336.18
10,001–50,000	68	11.5	1500–2500 g	5104.36
50,000–100,000	6	1	>2500 g	5622.63

**Table 4:** Impact of variables on duration of donation and amount of milk donated.

	Duration of Donation		Amount of Milk Donated	
Characteristic	Median (IQR)	*p*-Value	Median (IQR)	*p*-Value
**Working**		0.2		0.008
no	54 (30, 108)		1915 (808, 4083)	
yes	61 (30, 117)		2450 (1000, 6250)	
**Type of delivery**		0.8		0.022
cesarean	57 (27, 105)		2650 (1115, 6800)	
natural	60 (30, 113)		2000 (900, 4775)	
**Parity**		0.3		0.5
multiparous	61 (28, 112)		2200 (1000, 5200)	
primipara	56 (30, 95)		2000 (900, 5450)	
**Previous breastfeeding**		0.3		0.8
NO	56 (30, 104)		2100 (863, 5598)	
SI	64 (30, 116)		2150 (1000, 5000)	
	**Spearman Correlation**	***p*** **-Value**	**Spearman Correlation**	***p*** **-Value**
**Age**	0.19	<0.0001	0.18	<0.0001
**Birth Weight**	0.1	0.0174	−0.08	0.0618
**Gestational Age**	0.14	0.0009	−0.14	0.0008

## Data Availability

The original contributions presented in the study are included in the article, further inquiries can be directed to the corresponding author.
